# A practical guide to characterising ecological coexistence

**DOI:** 10.1111/brv.70079

**Published:** 2025-10-10

**Authors:** Adam T. Clark, Lauren G. Shoemaker, Jean‐François Arnoldi, György Barabás, Rachel Germain, Oscar Godoy, Lauren Hallett, Canan Karakoç, Serguei Saavedra, Sebastian J. Schreiber

**Affiliations:** ^1^ Department of Biology University of Graz Holteigasse 6 Graz 8010 Austria; ^2^ Botany Department University of Wyoming 1000 E. University Ave Laramie WY 82071 USA; ^3^ Centre National de la Recherche Scientifique Experimental and Theoretical Ecology Station 2 route du CNRS Moulis 09200 France; ^4^ Division of Biology Linköping University Linköping SE‐58183 Sweden; ^5^ Institute of Evolution, Centre for Ecological Research Konkoly‐Thege Miklós út 29‐33 Budapest H‐1121 Hungary; ^6^ Department of Zoology The University of British Columbia 6270 University Blvd #4200 Vancouver BC V6T1Z4 Canada; ^7^ Estación Biológica de Doñana (EBD‐CSIC) Americo Vespucio 26 Sevilla 41092 Spain; ^8^ Institute of Ecology and Evolution University of Oregon Onyx Bridge, 272, 1318 Franklin Blvd Eugene OR 97403 USA; ^9^ School of Biological Sciences and Center for Microbial Dynamics and Infection, Georgia Institute of Technology Atlanta North Avenue GA 30332 USA; ^10^ Department of Civil and Environmental Engineering, MIT 77 Massachusetts Avenue Cambridge MA 02139 USA; ^11^ Santa Fe Institute 1399 Hyde Park Rd Santa Fe NM 87501 USA; ^12^ Department of Evolution and Ecology and Center for Population Biology University of California Davis CA 95616 USA

**Keywords:** asymptotic return rate, ecological coexistence, empirically tractable, invasion growth rate, mutual invasibility, parameter sensitivity, structural stability, time to extinction

## Abstract

Coexistence is simultaneously one of the most fundamental concepts of ecology, and one of the most difficult to define. A particular challenge is that, despite a well‐developed body of research, several different schools of thought have developed over the past century, leading to multiple independent, and largely isolated, branches of literature with distinct methodologies. Here, we provide a broad overview of the most common concepts and metrics currently used to detect and characterise ecological coexistence. We first introduce four classes of behaviour, which jointly describe the ways in which community dynamics can unfold: (*i*) the existence of a feasible steady state (or invariant set), i.e. where all coexisting species retain positive abundances in the long‐term in the absence of interference by external forces; (*ii*) the existence of a local attractor that draws the community towards a feasible steady state from within a restricted set of starting conditions; (*iii*) the existence of a global attractor that draws the community towards feasible steady states from any non‐zero starting condition; and (*o*) a null transient state, where species abundances vary over time irrespective of steady states and attractors. Next, we explain how these classes of behaviour relate to commonly used metrics for identifying and characterising coexistence, including analyses of parameter sensitivity, asymptotic return rates, invasion growth rates, and time to extinction. We then discuss the scope and limitations of each of these behavioural classes and corresponding metrics, with a particular focus on applications in empirical systems. Finally, we provide a potential workflow for matching empirical questions to theoretical tools, and present a brief prospectus looking forward to opportunities for advancing and integrating research on coexistence.

## I. INTRODUCTION

Understanding when different combinations of species can coexist is one of the primary goals of ecology (MacArthur, 1972). Accurate prediction of coexistence is also a fundamental prerequisite for addressing many of the discipline's most pressing challenges, with potential applications including effective design of conservation and restoration strategies (Bradshaw, 1992), identifying and managing invasive species (Moles, Gruber & Bonser, 2008), estimating rates of biodiversity change and turnover (Newbold *et al*., 2015; Blowes *et al*., 2024), and forecasting impacts of global change (Sage, 2020; Usinowicz & Levine, 2018; Van Dyke, Levine & Kraft, 2022). However, despite almost a century of theoretical advances in our understanding of coexistence, the field remains largely fractured, resulting in multiple schools of thought with their own distinct definitions for what qualifies as coexistence, and surprisingly few attempts to reconcile these dominant frameworks and their corresponding metrics (Lawton, 1999; Donohue *et al*., 2016).

Within the coexistence literature, it has been especially challenging to synthesise insights about coexistence across empirical studies. This challenge arises due to both the literature's fragmentation in the definitions and metrics used to assess coexistence, and because existing metrics are often challenging to apply in real‐world contexts, requiring ample data, strong theoretical assumptions, and both empirical and analytical expertise (Levine *et al*., 2017; Clark, Hillebrand & Harpole, 2019; Spaak *et al*., 2023). Arguably as a consequence of this fragmentation and complexity, there are currently no cross‐system meta‐analyses or global studies of coexistence, and surprisingly little is known about how opportunities for coexistence compare across space, time, and species.

This review seeks to provide a high‐level overview of recent advances in coexistence theory, with the goal of summarising the disparate literatures for theoreticians and empiricists alike. Our scope encompasses all kinds of ecological communities and species interaction types, although as we will discuss, a focus on competitive interactions and sessile species tends to dominate the literature. Our primary aim is to explain the strengths and weaknesses of different approaches that have been developed to identify *whether coexistence is possible* within a particular site and system, with a focus on practical applications in real‐world settings. As this topic alone already covers a dauntingly large literature, we will largely neglect the more specific question of *how coexistence is maintained* – although see Section III.3 for a brief overview of some of the major classes of coexistence mechanisms, and suggestions for further reading.

We begin this review with a brief summary of the historical development of coexistence theory, and explain how it shaped the modern concept of coexistence (Section II). Next, we introduce the most common kinds of dynamic behaviour currently used by theorists to describe coexistence (Section III.1), and, correspondingly, the most common metrics used to identify these behaviours (Section III.2). We then provide a more detailed discussion of the general challenges that empirical systems pose for efforts to measure coexistence (Section IV.1). Finally, we present guidelines and a recommended workflow for matching empirical questions to theoretical tools (Section IV.2), and suggest potential ways forward for the practical study of coexistence (Section IV.3).

## II. HISTORY OF THEORETICAL DEVELOPMENT

Coexistence has been a central tenet of population and community ecological theory since the disciplines' earliest beginnings. Efforts to model community dynamics published, in rapid succession by Lotka (1925) and Volterra (1926), both discuss coexistence criteria, with Volterra (1926) in particular providing a detailed theoretical derivation of the necessary conditions for various forms of stable coexistence across different community structures (see Table 1 for glossary).

**Table 1:** Glossary.

**Coexistence**	The ability of a community of co‐occurring species to persist (i.e. retain positive abundances) across a defined set of spatial and temporal scales. Other published sources provide more specific definitions explained below, e.g. related to *steady states* or stability; however, these definitions also vary widely across sub‐fields and sources. To avoid ambiguity, we therefore refer to the specific kinds of dynamic behaviour described in Section III.1 when more precise definitions of coexistence are necessary.
**System state**	A measurement of the dynamic variables in a system at a given moment in time. In coexistence studies, states usually refer to the abundance or biomass of organisms within a community.
**Parameters**	Quantities that govern the dynamics of states according to an equation, such as a population model – e.g. intrinsic growth rates, carrying capacities, or species interaction coefficients. Unlike states, parameters are constant for a given set of conditions.
**Steady state (and invariant sets)**	A *steady state* describes a system state or set of states within which the system will remain bounded, at least in the absence of interference by external forces (i.e. processes that impact dynamic variables, but do not themselves fall within the scope of the system as defined by the researcher – common examples include extreme weather events or experimental disturbance treatments). Ecological studies often focus on the concept of equilibria – e.g. a set of species abundances that remain fixed at a single set of values over time. More generally, *invariant sets* include a wider range of dynamic behaviours, including, e.g. periodic and quasi‐periodic orbits, or chaotic motions. This broader definition is especially important for complex community dynamics such as predator–prey oscillations.
**Feasible**	In the ecological literature, feasible is used both to describe a state in which all species in a community have non‐negative abundances, or, alternatively, where they all have positive abundances (i.e. greater than zero; a self‐evident empirical requirement that is sometimes overlooked in models). Most recent literature uses the latter of these definitions. Unless otherwise stated, we use the term to refer to positive states in this review. Note, however, that a feasible state does not necessarily imply a steady state (or invariant set), nor is it necessarily stable, e.g. it need not be associated with an *attractor*.
**Attractor**	A steady state (or invariant set) to which a system will return following externally driven changes to the state variables (e.g. as observed with the classical concept of a stable equilibrium). For a *local attractor*, systems can only recover if perturbations are sufficiently small (determined by the size and properties of the attractor), whereas for a *global attractor*, all feasible starting states lead to the same steady state or invariant set. Permanence describes systems with global attractors where the corresponding steady state or invariant set is also feasible.
**Necessary and sufficient conditions**	For any theoretical outcome, necessary conditions must be met for the outcome to take place, but they do not guarantee that it will. By contrast, a sufficient condition guarantees that an outcome will take place, but does not necessarily need to be met in order for the outcome to occur. For example, the existence of a feasible equilibrium is necessary (but not sufficient) for permanence, whereas permanence is sufficient (but not necessary) for the existence of a feasible equilibrium (Hofbauer & Sigmund, 1998).

These early findings were immensely influential for subsequent studies of coexistence. In particular, Gause's empirical tests of the theories of Lotka (1925) and Volterra (1926) popularised the competitive exclusion principle, which hypothesised that in order to coexist, species needed to differ in terms of their biological needs or ecological niches (Gause, 1934). This hypothesis inspired a proliferation of empirical studies that sought to identify the biological factors that enabled coexistence for particular groups of species (e.g. MacArthur, 1958; Park, 1962; Paine, 1966). The hypothesis was formalised mathematically by Nicholson (1933), with a later generalisation by Levin (1970) who showed that for a large class of models, the number of species stably coexisting at equilibrium could not exceed the number of limiting factors (called “control factors” in Nicholson, 1933). These limiting factors are often interpreted as specific resources such as light or nitrogen, although Levin (1970) was careful to note that they could represent any combination of variables with independent effects on species' *per‐capita* growth rates (including, e.g. predators, spatial or temporal structure, etc.).

Levin (1970) and Nicholson (1933) showed that limiting factors were critical for understanding the conditions under which coexistence was possible – but also that identifying these factors in practice was likely to be “extremely difficult” for several reasons (Levin, 1970). First, biological systems include many different interacting species and environmental variables, and these variables also tend to be highly correlated – thereby complicating efforts to identify the number (Ellner, 1988) and identity (Abrams, 1988) of independent dimensions acting on growth. Similarly, for certain kinds of processes – e.g. self‐limitation – it can be difficult to measure and model impacts in terms of limiting factors (McPeek, 2012). Finally, because species often exhibit non‐linear growth responses to factors such as resource availability or the abundance of competitors, spatial or temporal variability can cause individual variables to act like multiple independent factors – e.g. with periods of high *versus* low variability leading to different average growth rates for different species (Levins, 1979; Armstrong & McGehee, 1980).

To overcome these challenges, two main paradigms emerged for studying coexistence while circumventing the need to identify explicit limiting factors: analyses of *asymptotic return rates* and of *invasion growth rates* (Turelli, 1978) (see Section III.2). Asymptotic return rates describe the tendency of systems to return to steady state following small perturbations, and have their origins in applied mathematics and physics. These rates can be applied to conduct local stability analysis and determine whether a system is likely to recover from small perturbations. Their use became popular in ecology following applications by MacArthur (1958, 1970, 1972) for analysing competitive interactions. The metric proved particularly effective for assessing coexistence in systems with many interacting species or resources (May & MacArthur, 1972; May, 1973) – especially in cases where coexistence emerged as a direct result of these interactions (Holt, 1977; Lawlor, 1979). An important finding of these studies was that asymptotic recovery in ecological communities is largely governed by the degree to which species interaction coefficients are linearly independent – i.e. such that no two species (or two combinations of multiple species) have exactly the same impacts on the rest of the community (MacArthur, 1970; Chesson, 1990). Importantly, this insight suggested that empirically measuring interaction strengths among species might be an effective way to predict and classify coexistence in real‐world settings (May & MacArthur, 1972).

Analysis of invasion growth rates yielded a practical metric for identifying coexistence that could be estimated analytically, e.g. from model simulations, or empirically, through invasion experiments (MacArthur & Levins, 1967; Turelli, 1978; Chesson & Warner, 1981). The general intuition behind invasion analysis is that if each species in a community can increase from rarity when all other species are at steady state, then all successfully invading species should generally be able to coexist. Initially, studies stressed that the validity of this approach as a test for stable coexistence had “not yet been proven” (Turelli, 1981, p. 5), and that they amounted to a “heuristic coexistence criterion” (Turelli, 1986, p. 331). Indeed, simply checking that invasion rates are positive for all species in a community does not guarantee coexistence, especially for species‐rich communities (Barabás, D'Andrea & Stump, 2018), although more mathematically rigorous analyses of those invasion rates can still be highly effective, e.g. *via* permanence theory (Hofbauer, 1981; Butler & Waltman, 1986; Schreiber, 2000) – see Section III.2.*c* for details. Analyses of invasion growth rates quickly gained in popularity, both because they often yielded simpler, more mathematically tractable predictions than analyses of asymptotic return rates, and because they were better able to account for effects of large disturbances and complex community dynamics, rather than just small perturbations around a static equilibrium (as is the focus of local stability analysis) (Turelli, 1980, 1981).

In particular, a ground‐breaking series of articles leveraged invasion analyses to catalogue the ways in which coexistence can arise as a result of environmental fluctuations across time (Chesson, 1994) and space (Chesson, 2000a). Chesson's theories (Chesson, 2000b, 2008, 2018) would eventually become the dominant framework used for explaining why species are able to coexist in spatially or temporally variable environments (see Section III.3) – to the point that later authors came to refer to his work as ‘Modern Coexistence Theory’ (Mayfield & Levine, 2010; HilleRisLambers *et al*., 2012; Grainger, Levine & Gilbert, 2019), in an apparent nod to the Modern Evolutionary Synthesis.

## III. CURRENT PARADIGMS AND METRICS

Although classical approaches for assessing coexistence are still commonly used by ecologists today, many of these methods have been refined to account better for important aspects of real‐world ecological systems (see Section IV.1). This methodological diversity has added important tractability and nuance to coexistence theory, but it also poses a problem: different methods define coexistence in distinct, and sometimes even contradictory ways – e.g. based on a community's ability to recover from small *versus* large disturbances (Turelli, 1978; Schreiber, 2006).

To compare and contrast these different methods, we first introduce four broad classes of dynamic behaviour on which ecologists tend to focus when studying coexistence (Section III.1). These behaviours roughly correspond to the different definitions of coexistence (or lack thereof) that are applied in contemporary theoretical studies. We then discuss metrics that are commonly used to identify and characterise each of these four behavioural regimes (Section III.2), as well as their scope and limitations. Challenges associated with applying these metrics in empirical systems are discussed in more detail in Section IV.

### (1) Classes of dynamic behaviour

**Fig. 1:**
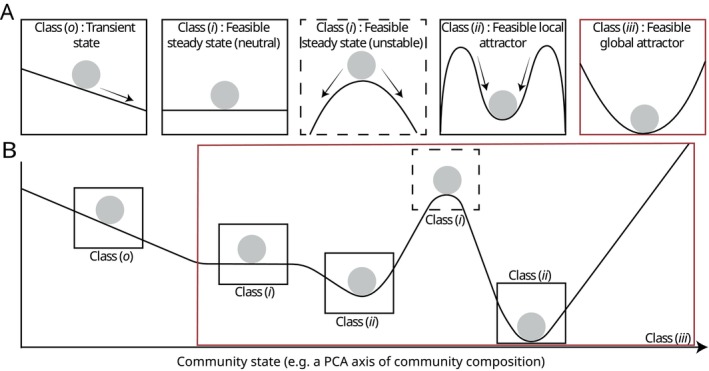
Four main classes of behaviour describing species abundance dynamics in the context of coexistence. (A) Conceptual illustration of abundance dynamics following each of the four behaviour classes discussed in Section III.1, using a ‘ball‐and‐cup’ metaphor. The position of the ball along the *x*‐axis represents the system state [e.g. the abundances of a set of species, as might be represented by a principal components analysis (PCA) axis], and the rolling trajectories in panels *o*–*iii* represent, respectively, a lack of steady state, the presence of a feasible steady state (including examples of both ‘neutrally’ stable and ‘unstable’ cases), a feasible local attractor, and a feasible global attractor. (B) An example dynamic landscape combining all four classes of behaviour as part of a single system. Note that dynamic behaviours within subsections of the system can follow different combinations of behaviours. The feasible steady states in class *i* plus the two feasible local attractors in class *ii* jointly make up the system's global attractor (class *iii*). In this example, in the long‐term, the system is drawn away from transience and towards at least one of these states from any feasible starting state, with the precise end‐state dependent on initial conditions.

Species dynamics can be broadly grouped into four main classes of nested behaviours (Fig. 1). These behaviours are: (*i*) feasible steady states (and feasible invariant sets) – systems where all species retain positive abundances, at least in the absence of interference by external forces; (*ii*) feasible local attractors – systems with local attractors that draw species towards feasible steady states given a specific neighbourhood of starting abundances; (*iii*) feasible global attractors – systems with a global attractor that draws species towards some set of feasible steady states or invariant set from any starting condition with non‐zero species abundances (potentially including multiple different local attractors); and, finally, (*o*) transient states – systems in which species abundances vary over time either without or prior to settling into a steady state that may or may not yield coexistence.

Each of these behaviours relates to somewhat different aspects of coexistence and stability. In practical terms for real‐world communities: a feasible steady state (*i*) implies that species will persist together in the long term so long as they remain undisturbed by external forces; a feasible local attractor (*ii*) implies that species can recover back to a steady state following small disturbances; a feasible global attractor (*iii*) implies that the community can recover even from large disturbances; and transient dynamics (*o*) imply that the system is in transition, and species will either eventually go extinct or the system's dynamic behaviour will switch to one of the three other regimes. Critically, in the absence of demographic stochasticity (see Section IV.1.*c* for definitions), behaviours *i*–*iii* also follow a partially nested hierarchy: a feasible steady state (or invariant set) is a necessary (but not sufficient) condition for a feasible local attractor, which is itself a necessary (but again, not sufficient) condition for a feasible global attractor. Below, we consider these behaviours in more detail.

#### (a) Feasible steady state

A feasible steady state describes conditions for which coexisting species' abundances remain at fixed positive values over time (when at an equilibrium) or constrained within a bounded set of positive values (for invariant sets more generally, e.g. limit cycles). A simple example accounting for two types of feasible steady states is a model with stable carrying capacity and a positive Allee threshold, below which the population tends towards extinction. Both the carrying capacity and the Allee threshold are feasible equilibria. However, the smallest disturbances from the Allee threshold could drive the population to extinction. Thus, although feasible steady states do not guarantee long‐term coexistence, the lack of a feasible steady state (or invariant set) implies either extinction of some species, or that the system will become transient as it moves towards some other dynamic regime.

Identifying feasible equilibria is often more analytically straightforward than applications of other coexistence metrics, and the existence of feasible equilibria can sometimes even be used to imply the existence of local or global attractors – leading some studies to use of feasibility as a stand‐alone proxy for coexistence (Saavedra *et al*., 2017; Grilli *et al*., 2017; Song, Rohr & Saavedra, 2018; see Section III.2.*a*). Note that although most studies of feasible coexistence have focused on analysing model equilibria, these approaches sometimes can be applied to more complex invariant sets as well – e.g. to identify ranges of model parameters that lead to oscillatory cycles or even chaos (Barabás, Meszéna & Ostling, 2012; Barabás & Ostling, 2013; Bunin, 2017; McCann & Yodzis, 1994).

#### (b) Feasible local attractor

If some range of starting conditions exists from which system dynamics are drawn towards a particular feasible steady state (or invariant set), then these states are said to be part of a local attractor. This range of starting conditions is called the ‘basin of attraction’ of the attractor, and is often visualised as valleys in classical ball‐and‐cup diagrams (Fig. 1). Feasible local attractors support coexistence by counteracting interference by external forces, such as small environmental perturbations, that might otherwise drive species away from a feasible steady state and towards extinction. Indeed, in the absence of an attractor, even arbitrarily small perturbations will, in the long term, drive species to extinction (Schreiber, 2006). Local attractors, however, only directly govern dynamics within their basin of attraction. Thus, the existence of a feasible local attractor does not necessarily guarantee long‐term coexistence – e.g. if initial abundances fall outside of the basin of attraction, or species are subjected to sufficiently strong perturbations, then long‐term abundance dynamics can be driven towards other system states, such as those associated with alternate community structures or even species extinction (Almaraz *et al*., 2024).

Characterising dynamics around steady states relies on quantifying asymptotic return rates and extrapolating whether dynamics are likely to remain in that state or to move towards another dynamic regime (Turelli, 1978). Early work by Lewontin (1969), Levin (1970), MacArthur (1970), and May (1973), for example, identified local attractors by computing the eigenvalues for the Jacobian matrix at model equilibria. These approaches are still popular today, and are discussed in more detail in Section III.2.*b*. For more general classes of invariant sets (e.g. oscillatory dynamics, chaos), local attractors can be identified by calculating return rates along the entirety of the system's dynamic trajectory, e.g. using Lyapunov exponents. These approaches are discussed in Section III.2.*b.i*.

#### (c) Feasible global attractor

In ecology, feasible global attractors refer to system dynamics in which a set of species is drawn towards feasible steady states (or feasible invariant sets) from any feasible starting abundance, thereby meeting the criteria for permanence (Schuster, Sigmund & Wolff, 1979; Sigmund & Schuster, 1984). Thus, a feasible global attractor is effectively a local attractor for which the basin of attraction includes all feasible starting conditions. Feasible global attractors therefore ensure long‐term coexistence even in the face of strong disturbances and major re‐mixing of a community. So long as perturbations do not push a species to an abundance of zero, they will always be able to recover in the long run. This strong form of coexistence comes at the cost of strict requirements that are necessary for feasible global attractors to exist. Indeed, their existence necessarily excludes several classes of behaviours that might be considered to be compatible with coexistence under other definitions. For example, systems subject to Allee effects – where species must exceed some minimum abundance to achieve positive growth (Jang, 2013) – preclude feasible global attractors, even if a feasible local attractor exists.

The especially broad scope of global attractors can also make identifying and testing for them difficult. Invasion analysis was therefore introduced as a comparatively tractable approach for identifying these kinds of attractors (Turelli, 1978; Hofbauer, 1981; Schreiber, 2000). The general idea behind invasion analysis is that if all species in a community can increase in abundance when rare, then the system should be able to recover from most kinds of major disturbances – and that this behaviour is, at the very least, consistent with the existence of a feasible global attractor, e.g. as discussed by Turelli (1981, 1986). The development of permanence theory in the early 1980s provided more rigorous mathematical justification for these approaches, summarising the circumstances under which invasion analyses are sufficient for proving the existence of a global attractor (Hofbauer, 1981; Butler & Waltman, 1986; Garay, 1989; Schreiber, 2000; Patel & Schreiber, 2018) (see Section III.2.*c*.*i* for a more detailed discussion of caveats and limitations). Analyses of invasion growth rates remain in wide use today and are discussed in more detail in Section III.2.*c*.

#### (d) Transient state

In ecology, transient states refer to abundance dynamics varying either in the absence of, or prior to reaching, a steady state or invariant set (Hastings, 2004; Fukami & Nakajima, 2011). Transient dynamics can be short or long lasting, and may or may not ultimately drive communities towards feasible states in the long run. In practice, it can be challenging to determine whether a particular dynamical trajectory is truly transient, is in a basin of attraction, or is even part of some more complex invariant set such as a limit cycle. Due to this ambiguity, categorising something as a transient state usually implies that no steady states or invariant sets that influence the current state have *yet* been identified, but often does not definitively exclude the possibility of their existence.

### (2) Metrics for characterising behaviours

**Fig. 2:**
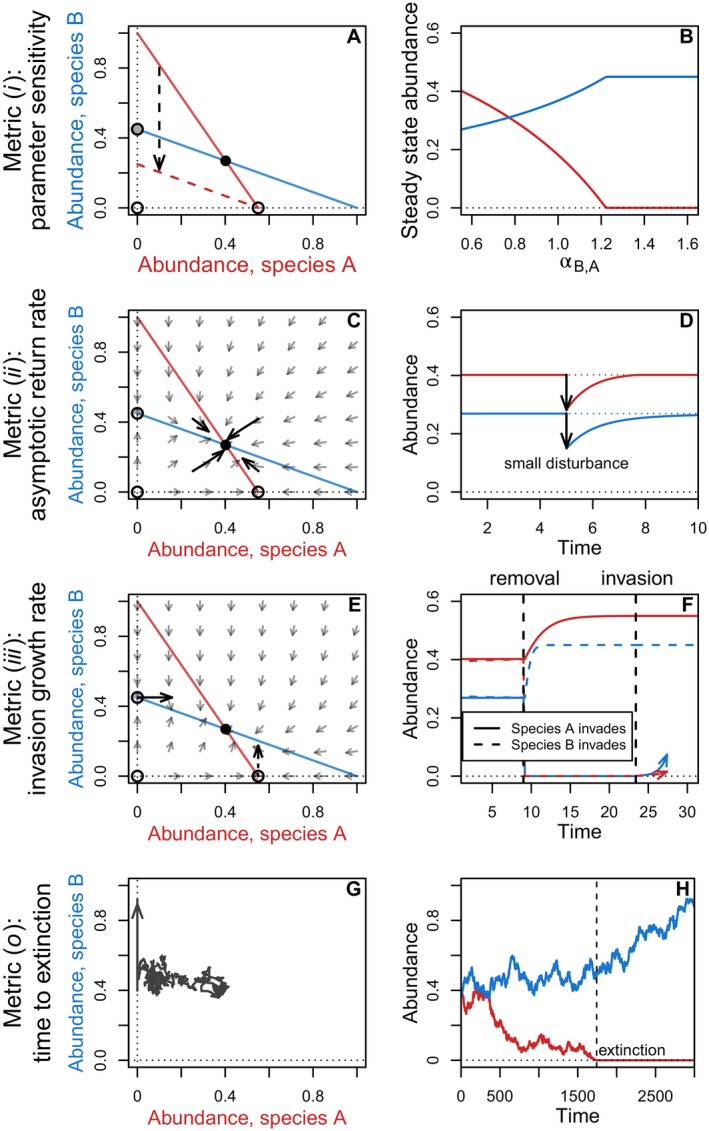
Conceptual illustration of the coexistence metrics described in Section III.2. Left column (A, C, E, G) shows phase diagrams. Axes show steady state abundances for each species; red and blue lines, respectively, show abundances at which species A and B experience zero net growth (zero net growth isoclines); empty circles show unstable equilibria; and filled circles show feasible attractors. Black arrows emphasise the main dynamics of interest for each metric. Right column shows corresponding impacts of the parameter change on steady state abundances (B) or time series of abundances (D, F, H) for each species. Time to extinction in G (o) is shown for a random walk with two species (thick black line and arrow). Other panels show results for the two‐species Lokta–Volterra competition equations. Parameter sensitivity in A and B (*i*) is demonstrated *via* changes in *α*_B,A_, which describes the impact of species B on the *per‐capita* growth rate of species A. The black dashed arrow shows the shift in the isocline associated with the parameter change, shifting the solid red isocline to the dashed red line, and causing a previously unstable single‐species equilibrium (with species B persisting) to become stable (light grey circle). Asymptotic return rates in C (*ii*) are demonstrated in response to a series of small perturbations around the feasible equilibrium – small grey arrows show the gradient of the system's dynamics, and the thick black arrows show return trajectories. Invasion growth rates in E (*iii*) are shown with solid and dashed arrows at the time of invasion, representing growth trajectories for a short time step for the invader and at low abundance for species A and B, respectively, with the competitor species at its single‐species carrying capacity.

Most contemporary studies rely on metrics that characterise specific aspects of coexistence, rather than focusing on general classes of dynamic behaviours themselves. The most widely used of these include: (*i*) parameter sensitivity – the quantification of how slight changes in parameter values alter system attributes, such as the existence and identity of species in feasible steady states; (*ii*) asymptotic return rate – the rate at which systems are drawn towards a particular steady state; (*iii*) invasion growth rate – the rate at which species are able to increase from low abundance; and (*o*) time to extinction – the average length of time for which species maintain positive population sizes. Importantly, each of these metrics relates to one or more of the dynamic behaviours discussed in Section III.1: parameter sensitivity is useful for assessing the robustness of steady states to uncertainty in, or perturbations to, parameter values; asymptotic return rates can be used to identify local attractors *via* local stability analysis; invasion growth rates can be applied to test for the existence of a feasible global attractor; and time to extinction can be calculated for any kind of abundance dynamic, including transient states that yield co‐occurrence on ecologically relevant timescales (Fig. 2). Below, we introduce these metrics in more detail, with a particular focus on how they are applied in practice.

#### (a) Parameter sensitivity

Parameter sensitivity is related to the general mathematical concept of ‘structural stability’, which focuses on whether the topological features of a dynamic system are preserved under small perturbations of its underlying equations or parameter values (Smale, 1967; Levin, 1970). Structural stability can be applied in studies of coexistence to examine the conditions under which global or local attractors can be maintained, or to identify critical points where small changes in parameters alter the system's stability, such as switching from an equilibrium to a periodic solution (Almaraz *et al*., 2024). In a growing body of ecological studies, however, the concept of structural stability has been defined somewhat more narrowly, to test whether small perturbations to model parameters lead to the loss of feasible steady states (Rohr, Saavedra & Bascompte, 2014).

Having grown out of this focus on steady states, analyses of parameter sensitivity in ecology typically take one of two approaches. The first focuses on the range of model parameter values within which feasible steady states can be maintained for a particular subset of species (May, 1973; Svirezhev & Logofet, 1983; Grilli *et al*., 2017; Saavedra *et al*., 2017; Saavedra, Medeiros & AlAdwani, 2020; Deng, Taylor & Saavedra, 2022; Allen‐Perkins *et al*., 2023). This approach provides a general overview of which regions of parameter space could potentially allow for coexistence, and which regions cannot. For example, under the Lotka–Volterra competition equations, if species A and B initially coexist, then increasing the competitive impact of species B on species A will eventually drive species A extinct (Fig. 2A, B). The second approach focuses on the localised effects of perturbations. It takes a known steady state, and examines the sensitivity of that steady state to small parameter perturbations (Vandermeer, 1970; Levins, 1974; Meszéna *et al*., 2006; Barabás *et al*., 2014). Steady states that are oversensitive to even very small changes in the parameters are not expected to persist for long, and are thus assumed not to allow for coexistence in the long term.

If the range of parameters allowing feasible steady states is large, those steady states are said to be ‘robust’ against parameter perturbations. This perspective has been especially effective at elucidating how different processes contribute to coexistence – e.g. generating qualitative insights about species' interactions and ecological niches that are independent of the specific model used to analyse the system (Meszéna *et al*., 2006; Barabás *et al*., 2014; Pásztor *et al*., 2016), quantifying the relative effects of pairwise *versus* indirect interactions on equilibria in the Lotka–Volterra competition equations (Saavedra *et al*., 2017; García‐Callejas, Bartomeus & Godoy, 2021), predicting which species have larger persistence times (Allen‐Perkins *et al*., 2023; Domínguez‐García *et al*., 2024), or dividing feasibility criteria into stabilising and equalising components (Godoy *et al*., 2018), analogous to classic partitions of invasion growth rates as discussed in Section III.3.

For equilibria, parameter sensitivity can be computed relatively easily – i.e. by identifying combinations of abundances and/or parameter values that lead to zero net population growth for all species in the community. However, parameter sensitivity can also be computed for more complex dynamics (Barabás *et al*., 2012, 2014; Barabás & Ostling, 2013), or even based on empirically observed time‐series data. For example, several recent studies have extended the scope of parameter sensitivity analyses to include effects of large perturbations (Tabi *et al*., 2020; Medeiros, Song & Saavedra, 2021), spatially and temporally structured environments (Saavedra *et al*., 2020; García‐Callejas *et al*., 2021; Luo *et al*., 2022; Song *et al*., 2023), and non‐linear conditions for specifying steady states (Cenci & Saavedra, 2018).

#### (b) Asymptotic return rate

Asymptotic return rates describe the long‐term response of system dynamics to infinitesimally small perturbations of system states by external forces. In ecological studies of coexistence, asymptotic return rates are usually applied to conduct local stability analyses that track the ability of species abundances to return to a particular steady state following small perturbations. If these return rates indicate that the system will always be drawn back towards steady state regardless of the direction of the small perturbation, the system is said to be asymptotically stable (Fig. 2C, D). Tests of asymptotic stability are analogous to testing for the existence of a local attractor – i.e. proving asymptotic stability is sufficient for identifying a local attractor. Thus, asymptotic stability of a feasible steady state implies coexistence, provided that species initial abundances fall within the basin of attraction of the local attractor, and that perturbations are sufficiently small that they do not push species out of this region.

For equilibria, asymptotic stability is tested by computing the eigenvalues of the Jacobian matrix around steady state. The details of this procedure are beyond the scope of this review [see Otto & Day (2011) for an excellent introduction], but in essence, eigenvalues summarise rates of change along a set of transformed axes (analogous to principal component axes), which make it easier to quantify net effects of different combinations of perturbations and species interactions. If the eigenvalues indicate that all species are drawn back towards equilibrium following small perturbations, then the system is asymptotically stable. Different indicator criteria must be used depending on the kind of system. For continuous‐time systems in which population dynamics play out smoothly over time, e.g. as might be expected for algae or bacteria, the return towards equilibrium occurs if the real part of the ‘leading’ eigenvalue (i.e. the eigenvalue with the largest real component) is negative. For discrete‐time systems in which population dynamics occur at regular intervals, e.g. as might be assumed for annual plants or some insects, the absolute value of the ‘dominant’ eigenvalue (the eigenvalue with the largest magnitude) must be less than 1. Several studies have also proposed methods for unifying the concepts of parameter sensitivity (see Section III.2.*a*) and asymptotic stability as part of a single analysis (Arnoldi & Haegeman, 2016; Song & Saavedra, 2018; Medeiros *et al*., 2021) – although these approaches are not yet in wide use.

There are several important considerations for interpreting asymptotic return rates. For example, assessing only a subset of eigenvalues, or focusing only on their sign but not their magnitude, can give a misleading picture of community dynamics as a whole. This is because species within a community can present different recovery dynamics. For example, the leading eigenvalue – which is often reported as a stand‐alone index of asymptotic stability – is primarily determined by the long‐term recovery rate for the slowest dynamic component of the system. Thus, even if only a single rare species fails to recover from a perturbation, the real part of the corresponding leading eigenvalue will still indicate a lack of asymptotic stability for the entire community, with no obvious indication of which species is responsible for the result, or what alternate states the community might shift to in response (Arnoldi, Loreau & Haegeman, 2016; Arnoldi *et al*., 2018). Similarly, this property can be misleading in systems that are subject to repeated perturbations, as the leading eigenvalue typically only includes information on long‐term responses, rather than short‐term dynamics (Arnoldi *et al*., 2018).

##### (i) Dynamic approaches for assessing return rates

Real‐world communities tend to be highly dynamic, which limits the utility of methods that focus on systems near equilibrium (Pimm *et al*., 2019). Although theoretical methods exist for analysing asymptotic stability in systems with more complex dynamics – e.g. that converge towards invariant sets such as limit cycles or quasi‐periodic orbits (Sell, 1966; Hirsch, Pugh & Shub, 1970; Breunung, 2022) – applying these metrics in empirical systems can be difficult. The problem is twofold. First, given an observed dynamic trajectory, it no longer suffices to test asymptotic return rates around a single fixed point – rather, return rates must be calculated along the entirety of that trajectory. Second, even if the observed part of a trajectory can be shown to be an attractor such that nearby states will always converge to it, there is no guarantee that the trajectory corresponds to long‐term coexistence. As an extreme (although empirically common) example, dynamic trajectories for a logistic growth model with growth rate *r* < 0 and starting population density *N* below the carrying capacity (i.e. *N < K*) will converge from any feasible starting abundance, but that convergence will be towards extinction rather than coexistence.

To address these challenges, an increasingly common approach is to apply forecasting tools such as empirical dynamic modelling (EDM) to approximate community dynamics, and then analyse the stability of the resulting model (Sugihara, 1994; Deyle *et al*., 2016). EDM uses time‐series observations to fit a series of piecewise locally weighted linear regressions that jointly describe how abundance dynamics, interactions among species, and impacts of environmental covariates change over time. Estimates of the Jacobian matrix describing community dynamics at each time step can then be computed directly from these piecewise regressions. Asymptotic stability is then tested in one of two ways: either individual estimates of the Jacobian matrix are used to identify a potential equilibrium towards which the system is being drawn (Ushio *et al*., 2018), or the full set of Jacobian matrices can be applied to approximate their corresponding Lyapunov spectrum (effectively a time‐varying generalisation of eigenvalues, which describe return rates along a dynamic trajectory; Oseledec, 1968). In addition to assessing asymptotic return rates, these time‐varying estimates of the Jacobian matrix can also be used to assess parameter sensitivity, as discussed in Section III.2.*a* (Cenci & Saavedra, 2019). For a more detailed review of EDM, including a discussion of available software for carrying out analyses, see Munch, Rogers & Sugihara (2023). For a more general discussion of local stability analysis along dynamic trajectories, see Medeiros *et al*. (2025).

#### (c) Invasion growth rates

Invasion growth rates quantify the average *per‐capita* growth rate of a species when it is relatively rare (termed the ‘invader’), and the rest of the community is at steady state (termed the ‘resident’). Intuitively, if the invasion growth rate of a species is positive, the species can increase from rarity and, thereby, escape extinction risk at least in the short term (Case, 1995; Arnoldi *et al*., 2022). Alternatively, if the invasion growth rate of a species is negative, then once a species reaches low abundance it would decline to extinction under current conditions. In the mathematical literature, invasion growth rates have also been used to characterise whether coexistence occurs in the sense of a feasible global attractor [also known as permanence or uniform persistence (Hofbauer, 1981; Schreiber, 2000; Patel & Schreiber, 2018)]. Only recently have the more heuristic approaches in the ecological literature and the rigorous approaches in the mathematical literature begun to merge, providing an unique opportunity to evaluate simultaneously whether coexistence in the sense of permanence occurs in a mathematically rigorous manner, and to probe the mechanisms underlying this coexistence in ecologically meaningful ways.

In the empirical literature, invasion growth rates are often used to test the ‘mutual invasibility’ criterion. In its simplest form, invasion growth rates are quantified for each of two interacting species, and the mutual invasibility criterion is met if both species' invasion rates are positive (Fig. 2E, F). The mutual invasibility criterion has been used extensively to assess scenarios that yield coexistence (Chesson, 1994, 2018; Adler, HilleRisLambers & Levine, 2007; Barabás *et al*., 2018; Ellner *et al*., 2020), and has been extended to communities composed of more than two species by assuming that whenever a species becomes rare, the remaining species will approach a feasible steady state. Provided that invasion growth rates are positive for all species, coexistence at a feasible global attractor is (potentially erroneously) assumed to occur; however as we discuss below, mutual invasibility is necessary, but not sufficient, for proving this kind of coexistence.

The mutual invasibility framework has several advantages. First, as long as the removal of the invader species does not incite any co‐extinctions of any resident species, invasion analysis effectively tests whether each species in a community can successfully re‐establish itself from low density after being driven locally extinct. For this reason, mutual invasibility is often presented as a more biologically meaningful metric in systems that are subject to strong stochastic influences or frequent large disturbances (Turelli, 1981). Indeed, mathematical theory for coexistence in the face of environmental stochasticity relies almost exclusively on this metric (Schreiber, Benaïm & Atchadé, 2011; Hening & Nguyen, 2018; Benaïm & Schreiber, 2019). Second, because mutual invasibility focuses on system dynamics when the focal species is effectively absent from the community, invasion analysis can (at least in theory) ignore feedbacks between the invading species and the rest of the community, which can simplify mathematical analyses considerably, especially if the total number of species in the community is small. Third, the mutual invasion criterion readily lends itself to testing for mechanisms that maintain coexistence, facilitating understanding for not only *when* coexistence occurs, but also *how* coexistence is maintained, with a focus on the role of spatial and temporal variation in the environment (Chesson, 1994, 2018; Barabás *et al*., 2018; Ellner *et al*., 2020) (see Section III.3).

Species invasion rates can, in theory, be estimated directly from experiments where individual species are introduced at low density, or where the growth of invader species is evaluated along a density gradient of residents (Godoy & Levine, 2014; Kraft, Godoy & Levine, 2015; Wainwright *et al*., 2019). In practice, however, most studies instead use observations to parameterise theoretical models, and use these models to derive estimates of ‘niche differences’ (i.e. stabilising mechanisms, which quantify the degree to which species limit themselves compared to others) and ‘average fitness differences’ (i.e. equalising mechanisms, which quantify how competitive ability differs among species) (Chesson, 1990, 2008; Barabás *et al*., 2018; Buche *et al*., 2022; Spaak *et al*., 2023). The ratio of niche and fitness differences can then be used to assess whether invasion rates are expected to be positive for all species (i.e. if niche differences are sufficiently large enough to overcome fitness differences between species pairs (Adler *et al*., 2007) – see Section III.3).

Note that the concept of mutual invasibility also includes important parallels to the field of adaptive dynamics. In this framework, invasion criteria are often expressed as the sign of the invasion fitness of a rare mutant in the environment set by a resident *x*, denoted *s*_*x*_(*y*) (Geritz & Gyllenberg, 2005). When *s*_*x*_(*y*) > 0, the mutant can spread (although demographic stochasticity may still prevent establishment), while *s*_*x*_(*y*) < 0 ensures its extinction. This framing parallels the ecological concept of invasion growth rates, which similarly assesses the potential of a species to increase from rarity (see also Case, 1995; Arnoldi *et al*., 2022).

##### (i) Permanence theory

Although the mutual invasibility criterion is useful for understanding coexistence of a pair of species, its applicability to more diverse communities and non‐competitive communities is less clear. A common approach in the literature is to assume that, for a community of *N* species, all subcommunities with *N*–1 species coexist, and to require that the missing species in each subcommunity has a positive invasion growth rate (Chesson, 1994, 2018; Grainger *et al*., 2019; Ellner *et al*., 2019). However, there are two issues with this approach (Spaak & Schreiber, 2023). Mathematically, this condition is not sufficient to ensure coexistence in the sense of a feasible global attractor. More importantly, for most systems, whether in nature or in theory, not all subsets of *N*–1 species will coexist. Notably, in multitrophic systems, predators cannot coexist without their prey. Hence, this raises the question: when and how can one use invasion growth rates to characterise coexistence for more realistic, diverse communities?

While model‐specific solutions have addressed these concerns in some cases (e.g. Chesson & Kuang, 2008; Ke & Wan, 2020; Song & Spaak, 2024), the mathematical theory of permanence (i.e. the existence of a feasible global attractor) provides a general approach for addressing these kinds of questions. Although the general abstract mathematical theory was developed in the 1980s (Hutson, 1984; Butler, Freedman & Waltman, 1986; Hofbauer & So, 1989), its connection to invasion growth rates was not established until over a decade later (Schreiber, 2000; Garay & Hofbauer, 2003; Hofbauer & Schreiber, 2010). Despite this connection, this mathematical theory relied on advanced techniques from dynamic systems theory (e.g. Morse decompositions, ergodic theory) and, consequently, had limited impact on coexistence research.

**Fig. 3:**
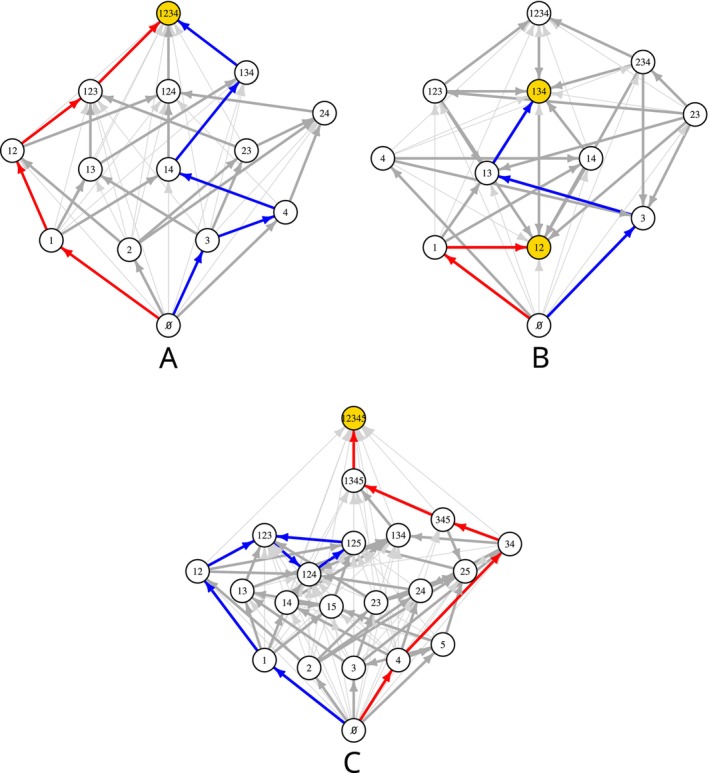
Invasion graphs for three empirically parameterised Lotka–Volterra models. (A) Acyclic invasion graph, where, as all subcommunities are invadable, the entire community is permanent (gold vertex). (B) Acyclic invasion graph but with two uninvadable subcommunities (gold vertices) which are attractors with missing species and, consequently, the community as a whole is not permanent. (C) Cyclic invasion graph with all subcommunities invadable, but requiring verification of additional conditions (see Schreiber, 2000) to ensure permanence of the entire community (gold vertex). The vertices in the graphs correspond to steady states of the Lotka–Volterra model and the directed edges correspond to potential community trajectories connecting the steady states as identified by invasion growth rates. Thick edges correspond to transitions due to single‐species invasions, while thin edges correspond to transitions due to multiple‐species invasions. The coloured edges indicate sequences of single‐species invasions terminating at an attractor for the model.

To help demystify this work, Hofbauer & Schreiber (2022) introduced invasion graphs as a means to verify the existence of Morse decompositions (Fig. 3). Using invasion growth rates, these graphs identify all potential community trajectories connecting steady states (or invariant sets) for different communities. As these trajectories may correspond to single‐ or multiple‐species invasions, invasion graphs differ fundamentally from community assembly graphs (e.g. Law & Morton, 1996), which only consider single‐species invasions, do not characterise community trajectories connecting distinct communities, and consequently, do not provide a means for verifying permanence.

Hofbauer & Schreiber (2022) showed that as long as the invasion graph has no cycles (i.e. no ‘rock–paper–scissor’ like dynamics), coexistence occurs if and only if, for every steady state with one or more missing species, at least one missing species has a positive invasion growth rate (Fig. 3A,B). Hence, in the absence of cycles, the sign of the invasion growth rates fully determines whether coexistence *via* a global attractor occurs. Alternatively, when the invasion graph has a cycle, one needs to analyse the magnitudes of the invasion growth rates to identify whether the cycle is repelling and, consequently, whether the community has a feasible global attractor (Fig. 3C). This can be done using average Lyapunov functions (Hofbauer, 1981), Poincaré maps (Krupa & Melbourne, 1995), or ergodic theory (Schreiber, 2000).

#### (d) Time to extinction

Even in transient systems where coexistence is not predicted, co‐occurrence of species can still take place over ecologically meaningful timescales (Lewontin & Cohen, 1969; Turelli, 1980). Time to extinction provides a quantitative measurement of this tendency, with longer time to extinction indicating longer periods of transient coexistence before extinction occurs (Fig. 2G,H). An advantage of this metric is that it can be applied across ecological systems, regardless of their dynamic behaviour, making it especially useful for characterising transient dynamics in which other coexistence metrics do not apply, or in systems where little is known about underlying steady states and attractors.

Extinctions can be driven by both deterministic processes (e.g. *via* declines in average resource concentrations or habitat size, or loss of obligate mutualists), and by random chance (e.g. stochastic fluctuations in demographic rates, or in environmental conditions). At least in theory, the timing of extinctions resulting from deterministic dynamics can be forecast exactly as a function of observed system states and dynamics. However, most modelling frameworks require additional considerations – e.g. in dynamic systems models, abundances approach zero asymptotically, such that they come infinitely close to, but never quite reach, extinction. Common solutions to this problem include applying an extinction cut‐off (quasi‐extinction) at an arbitrarily small population size, or including a stochastic component when modelling small populations (Holmes *et al*., 2007). For models where extinctions result from random fluctuations, time to extinction is typically reported in terms of an expected waiting time (i.e. the average time to extinction that might be observed over many repeated trials). Similar approaches can be used for examining expected time to extinction with demographic stochasticity, where demographic stochasticity can decrease time to extinction, especially for small population sizes (Lande, 1993; see Section IV.1.*c*).

Given relatively simple dynamics and strong theoretical assumptions, mean time to extinction can often be computed using analytical approximations (Lande & Orzack, 1988). For more complex systems, it can often be more efficient to estimate time to extinction directly through long‐term simulations of a fitted model (van Nes & Scheffer, 2004; Schreiber *et al*., 2023). Simulation‐based approaches for studying time to extinction are especially well developed in population ecology, where carefully tuned integrated population models and integral projection models (IPMs) can be applied to forecast population dynamics accurately for well‐studied species (Plard *et al*., 2019). Recently, more general methods have been developed that attempt to make similar forecasts using less data and fewer biological assumptions, e.g. based on insights from statistical mechanics (Arani *et al*., 2021) and delay‐embedding approaches (Clark *et al*., 2022).

### (3) Quantifying mechanisms that promote coexistence

While our focus is on methods for detecting when species coexist, a parallel and highly complementary body of work focuses on explaining how coexistence occurs – in other words, studies that quantify the strength of mechanisms that promote, or alternatively hinder, coexistence [reviewed by Palmer (1994), Vellend (2016), Chesson (2000b), McPeek (2022), and Wright (2022)]. Broadly, mechanisms that promote coexistence allow species to differentiate from one another in their degree of niche overlap, yielding differences in the strength of intra‐ and interspecific density dependence (Adler *et al*., 2018). These mechanisms fall into two broad categories: fluctuation‐independent mechanisms, which stabilise coexistence without requiring variation in environmental conditions, and fluctuation‐dependent mechanisms that necessitate variation in the environment across space and/or through time. While an in‐depth review of these mechanisms falls outside of our primary focus on assessing when coexistence occurs, we briefly highlight the prominent literature on mechanisms of coexistence below. We note that multiple mechanisms may often be at play in a given ecosystem (Wright, 2022; Stump & Chesson, 2015).

#### (a) Resource partitioning

When species partition their resource requirements, they can coexist, even under spatially and temporally homogenous environmental conditions. The resource ratio hypothesis, commonly referred to as R* theory, states that, given a single limiting resource, whichever species can persist at the lower resource equilibrium level (e.g. R*) will outcompete all other species (Volterra, 1926, MacArthur, 1972; Tilman, 1977, 1980, 1982b). Extending to multiple resources, *n* species can coexist on *n* limiting resources within a single spatially and temporally homogeneous site if, for each species, there exists a resource for which it has the lowest R*, suggesting that coexistence can be promoted by differences among species in terms of which resource is most limiting for their growth (assuming ‘essential resources’, and that species' consumption vectors and resource supply points meet certain requirements – see Tilman, 1982b). As explained in Section III.3.*c*, note that many more than *n* species can coexist on *n* limiting resources if sites are spatially or temporally heterogeneous – for a discussion of the cruicial role that tradeoffs can play in promoting such coexistence, see Tilman (2011). Since its formalisation, the resource ratio hypothesis has been experimentally tested, with strong support, although mostly in select grassland and phytoplankton systems due to logistical constraints (reviewed in Miller *et al*., 2005).

#### (b) Natural enemies

Specialisation of natural enemies, such as herbivores, predators, and pathogens, provides a top‐down mechanism that can promote coexistence independent of environmental fluctuations. This idea was popularised *via* the Janzen–Connell hypothesis, originally proposed as a mechanism for explaining high tree diversity in tropical systems (Janzen, 1970; Connell, 1971). The Janzen–Connell hypothesis posits that natural enemies can maintain coexistence by disproportionately affecting abundant species. As species increase in abundance, so too do their natural enemies. When enemies are specialised, this then creates a demographic (e.g. recruitment, seedling survival) advantage for rare species, as their natural enemies will correspondingly be rare, yielding strong conspecific negative density dependence (Terborgh, 2012). Partitioning of predators and natural enemies has been demonstrated empirically not only in the tropics, but also in temperate grasslands and forests, and even marine systems (reviewed in Terborgh, 2020).

#### (c) Spatial and temporal environmental variability

Variability in environmental conditions through space or time can increase available niche space, promoting coexistence of competing species. This idea dates back decades (MacArthur, 1958; Armstrong & McGehee, 1980; May & MacArthur, 1972; Turelli, 1978; Levins, 1979), but was formalised into a popular mathematical framework by Chesson (1990) for temporally variable (Chesson, 1994) and for spatially variable (Chesson, 2000a) environments. Broadly, environmental variability can promote coexistence even when fluctuation‐independent mechanisms may yield competitive exclusion. This can occur *via* multiple potential fluctuation‐dependent mechanisms: (*i*) the storage effect, where species partition environmental variation, and time periods or locations with beneficial environmental conditions correspond with reduced competition; (*ii*) relative non‐linearity, where species differ in their functional response to a shared resource that they compete for; and (*iii*) growth–density covariance (i.e. ‘habitat partitioning’ which only applies for spatial variation), which promotes coexistence if species aggregate in regions where they have high growth rates (Chesson, 2000b, 2018; Barabás *et al*., 2018; Ellner *et al*., 2019). A recent simulation‐based extension to this approach allows for alternative formalisation of mechanisms that promote coexistence under variable conditions, in essence by simulating invasion growth rates for each species under scenarios where spatial or temporal structure is disrupted (Ellner *et al*., 2019). The relative importance and strength of different coexistence mechanisms can then be quantified by comparing invasion rates with and without each structuring aspect (e.g. spatial or temporal heterogeneity), broadly categorising mechanisms based on resource availability (Letten *et al*., 2018), environmental–competitive mechanisms (Hallett *et al*., 2019, 2023; Aoyama *et al*., 2022), trait differences (Ellner *et al*., 2019), top‐down and bottom‐up forces (Shoemaker *et al*., 2020), or alternative frameworks.

## IV. RECONCILING THEORY AND PRACTICE

Recent coexistence research has made great strides in developing effective strategies to bring coexistence theory and practical applications into greater harmony. In the following sections, we first discuss key challenges in integrating coexistence theory with empirical tests. We then present some general guidelines and a recommended workflow for applying the coexistence metrics discussed in Section III.2 to characterise dynamic behaviour in empirical contexts. Finally, we end with a brief prospectus, in which we outline ongoing challenges in empirical coexistence research, and suggest potential ways forward.

### (1) Special challenges in empirical systems

Empirical systems are typically highly complex, diverse, and interconnected across space and time. Moreover, ecologists have limited *a priori* knowledge about the species, environments, and underlying biological processes that structure real‐world systems. Consequently, in addition to the theoretical caveats and scope limitations associated with each of the metrics discussed above, empirical systems present several general classes of challenges that must be considered regardless of the metric applied. These challenges are discussed in detail below, and include: the underlying biology of empirical systems is often poorly understood; empirical systems tend to be measured with large sampling uncertainty and are subject to both demographic and environmental stochasticity; the spatial and temporal scale of empirical observations are usually highly constrained; and key ecological properties of empirical systems often violate assumptions that are necessary in analyses of theoretical models.

#### (a) Incomplete biological understanding

There is currently no generally agreed upon standard model for describing dynamics in ecological systems. All ecological models must, therefore, be thought of as simplified abstractions, which are at best accurate within a limited scope of times, places, and conditions (MacArthur, 1970; Levin, 1992). The same caveat is inherited by any coexistence metric applied in empirical contexts: coexistence criteria can be tested given a particular set of theoretical assumptions, but there is no guarantee that resulting insights can be transferred to any given empirical system. Estimates of time‐to‐extinction forecasts illustrate this point particularly well. In a theoretical model, average extinction times can usually be estimated either through analytical formulae, or by simulation (Lande, Engen & Sæther, 1998; Arani *et al*., 2021; Schreiber *et al*., 2023). When applied in practice, however, these forecasts can fail for any number of reasons – e.g. because the wrong equations are chosen to represent the system, the model is improperly parameterised, or simply because initial conditions are imperfectly characterised (Auger‐Méthé *et al*., 2016; Plard *et al*., 2019; Rogers, Johnson & Munch, 2022). Moreover, even if model predictions perform well for a particular community and context, there is no guarantee that they will continue to do so for other times, places, and species (Carpenter *et al*., 2001).

Analogous issues exist for all other coexistence metrics. Parameter sensitivity analyses, for example, usually focus either on the steady states that are implied by a particular theoretical model (Saavedra *et al*., 2017), or on local approximations of these states expanded around an observed dynamic trajectory (Cenci & Saavedra, 2019). Similarly, although local stability analysis can be calculated with few assumptions about underlying system dynamics or governing equations (Deyle *et al*., 2016), these estimates are necessarily only accurate around the specific equilibria under consideration. Thus, changes to the system state (e.g. movement away from equilibrium due to large perturbations) or changes to underlying system dynamics (e.g. due to community turnover or environmental variability) will also lead to changes in the corresponding coexistence metrics (Tilman, 1982a).

At least in theory, analyses of invasion growth rates can be generalised across a wider range of system states than is true for other metrics. Nevertheless, empirical estimates of species invasion rates have been shown to change dramatically and largely unpredictably across environmental conditions (Matías *et al*., 2018; Germain, Mayfield & Gilbert, 2018; Hallett *et al*., 2019; Wainwright *et al*., 2019; Van Dyke *et al*., 2022). Thus, observing that a species fails to invade a community when introduced at low abundance might be indicative of a failure to coexist, or it could simply be a function of the specific conditions that were tested, and with a finite amount of empirical data there is no way to know for sure. While these uncertainties can usually be accounted for in analyses, doing so requires making strong theoretical assumptions about underlying dynamics and model functional forms (Letten, Ke & Fukami, 2017; Spaak *et al*., 2023; Weiss‐Lehman *et al*., 2022).

#### (b) Observation and detection errors

Empirical observations in ecology are notoriously noisy, leading both to high rates of observation error (e.g. differences between true *versus* measured species abundances), and detection errors (i.e. failing to detect species or incorrectly classifying species identities). Both of these kinds of error can have major ramifications for studies of coexistence. Most obviously, detection error can bias estimates of time to extinction, asymptotic return rates, and invasion success (Kindsvater *et al*., 2018; Dornelas *et al*., 2019). For example, if a species is classified as going extinct even though it is still present in the community, extinction rates and occurrences will be underestimated – or, alternatively, if surveys fail to detect small populations where extinction times are faster, then extinction estimates will be too high (Kuczynski, Ontiveros & Hillebrand, 2023). Many methods have been developed to help reduce such biases (Shimadzu, Foster & Darnell, 2016), although again, these tend to require large amounts of data, or strong assumptions about species dynamics and error structure.

Even if all species in a community are correctly detected and identified, observation error in abundance estimates can still confound analyses. For example, even small errors in abundance measurements can lead to large biases in model parameters (Bowler *et al*., 2022), as well as in corresponding estimates of coexistence metrics (Clark & Neuhauser, 2018). Particularly strong biases can occur when observation error is large relative to species average abundances. For example, if invasion rates are calculated using ratios, then measurement error can lead to estimates that have no defined mean or variance, which makes them exceedingly difficult to parameterise from empirical data (Marsaglia, 2006). Similar problems can occur for any kind of ratio distribution where noise is large relative to the quantity being observed – for example, when estimating interaction coefficients based on species' performance in mixture relative to monoculture (Carrara *et al*., 2015), or even when calculating estimates of species relative abundances or density.

#### (c) Environmental and demographic stochasticity

Thus far, we have focused primarily on deterministic models of species interactions. However ecological systems always experience extrinsic and intrinsic noise, making it unclear how well deterministic expectations match the realities of empirical, noisy systems. Stochasticity itself can add structure to systems (Shoemaker & Melbourne, 2016), changing dynamics such that systems that are predicted to coexist deterministically might not (Bowler *et al*., 2022), or alternatively stochasticity can yield coexistence when deterministic predictions predict competitive exclusion (Miller, Roxburgh & Shea, 2012). These impacts are typically grouped into two categories, known as environmental and demographic stochasticity, respectively (Lande, Engen & Saether, 2003; Schreiber, 2017). Environmental stochasticity arises from stochastic fluctuations in demographic rates due to stochastic fluctuations in environmental conditions such as temperature, precipitation, or nutrient availability. By contrast, demographic stochasticity arises from populations consisting of a finite and discrete number of individuals whose demographic fates are not perfectly correlated – e.g. independent coin flips determine whether each individual survives, grows, or reproduces.

Models with environmental stochasticity share many properties with their deterministic analogues (Levins, 1979; Chesson & Ellner, 1989; Chesson, 1994; Schreiber *et al*., 2011; Hening & Nguyen, 2018; Benaïm & Schreiber, 2019). The stochastic analogue of a feasible steady state is a feasible stationary distribution that describes, in the long term, the fraction of time spent near any feasible state (Schreiber *et al*., 2011; Hening & Nguyen, 2018; Benaïm & Schreiber, 2019). Unlike deterministic models, however, these stationary distributions are typically stochastic attractors – i.e. stationary distributions that system states converge towards over time. For example, if environmental stochasticity is added to a Lotka–Volterra competition model with alternate stable states, there is no ‘unstable’ stationary distribution that separates exclusion of one species from another. Instead, any feasible initial state may lead to the loss of either species with positive probability (Schreiber, 2021; Hening, Nguyen & Schreiber, 2022). The stochastic analogue of a feasible global steady state is known as stochastic persistence. Invasion growth rates are a key metric for identifying whether or not stochastic persistence occurs (Schreiber *et al*., 2011; Hening & Nguyen, 2018; Benaïm & Schreiber, 2019). Indeed, the use of invasion growth rates in coexistence theory for these stochastic models was largely popularised by Chesson's work on environmental stochasticity (Chesson & Warner, 1981; Chesson, 1994).

For models with environmental stochasticity, extinction typically only occurs asymptotically as population densities approach zero (Hening & Nguyen, 2018; Benaïm & Schreiber, 2019). Hence, extinction risk is typically measured by introducing a quasi‐extinction threshold below which the species is considered effectively extinct (Fieberg & Ellner, 2000). In sharp contrast, extinction typically occurs in finite time for models accounting for demographic stochasticity (Adler & Drake, 2008; Schreiber, 2017). Hence, coexistence is always transient in models with demographic stochasticity, regardless of the system's dynamic behaviour. However, these transients may be exceptionally long and well described by mean field models which average out the effects of demographic stochasticity. In particular, when a mean field model has a feasible local attractor, the time to extinction increases exponentially with community size (Faure & Schreiber, 2014; Schreiber, 2017). Hence, extinction risk can often be safely ignored for sufficiently large populations (Schreiber *et al*., 2023). By contrast, if the mean field models lack feasible local attractors, impacts of demographic fluctuations (i.e. random variability in birth and death rates) mount over time, such that time to extinction can be ecologically relevant even for large populations (Faure & Schreiber, 2014; Schreiber *et al*., 2023).

The simultaneous effects of environmental and demographic stochasticity are complex and only beginning to be understood. For example, even if invasion growth rates are positive for the mean field model (i.e. after averaging out the effects of demographic stochasticity), long periods of unfavourable environmental conditions can generate negative transients in the *per‐capita* growth rates of rare species. Under these situations, coexistence times only scale as a power function of community size (Ellner *et al*., 2020; Prodhomme & Strickler, 2024). Hence, even communities with large population sizes may be highly vulnerable to extinction. For these situations, positive invasion growth rates can be a poor metric of coexistence times. For example, Dean & Shnerb (2020) and Pande *et al*. (2020) showed that increasing environmental stochasticity can simultaneously make invasion growth rates more positive, yet simultaneously shorten coexistence times; a similar phenomenon occurs in models of competing species with Allee effects (Schreiber, Yamamichi & Strauss, 2019).

#### (d) The problem of scale

One of the most pervasive challenges in ecology is that of scale dependence – i.e. that the processes that drive ecological dynamics vary substantially across space, time, and contexts (Levin, 1992). These cross‐scale problems typically manifest in coexistence studies as a result of differences between the scales at which systems are observed and experimentally manipulated *versus* the scales that are most relevant for coexistence (Chesson, 2000b). As a simple illustration, consider the random walk model in Fig. 2G,H. The relative abundances of species in this model are entirely determined by demographic stochasticity – thus, at the global scale (i.e. considering all individuals in the simulation), all but one species will eventually drift to extinction. However, the system can appear to be both asymptotically stable and mutually invadable at smaller observational scales due to mass effects (Hubbell, 2001; Clark *et al*., 2019) – perturbations that reduce local species abundances below the average landscape‐level abundance are counteracted by immigration from outside of the local patch, and perturbations that increase local abundances above the average landscape level are counteracted because immigration from outside the patch is slower than within‐patch mortality.

Similar problems arise for many other kinds of ecological processes and coexistence metrics, although the underlying mechanisms are often more difficult to identify and compensate for. For example, invasion analysis can be challenging to implement using field experiments, as most theoretical frameworks require that invasion rates are averaged across the full range of spatial and temporal variability experienced by the community, whereas most experiments are carried out across a limited range of relatively homogeneous spatial replicates over just a few years. Thus, it can be unclear whether results from such experiments are indicative of real biological phenomena, or whether they reflect the limited range of conditions that were tested (Kraft *et al*., 2015). Moreover, these problems cannot be alleviated by simply increasing the scale at which observations and experiments are conducted – e.g. increasing the spatial grain of observations (e.g. the size of a plot) necessarily also destroys information about smaller‐scale spatial structure, which can lead to incorrect assessments of coexistence (Clark *et al*., 2019). Indeed, it seems likely that different aspects of coexistence (e.g. for different species, or different temporal periods) are driven by processes that act across many different scales, thereby requiring measurements across many different observational perspectives to capture their effects accurately (MacArthur, 1972).

#### (e) Violations of theoretical assumptions

Aspects of real‐world ecological systems can clash in important ways with common assumptions that are made to simplify theoretical analyses. For example, many empirical systems lack equilibria (DeAngelis & Waterhouse, 1987), and some appear to lack any kind of discernible steady state or invariant set at all (Pimm *et al*., 2019) – thereby limiting the kinds of coexistence metrics that can be computed. Even in systems that include feasible steady states, complex dynamics can impede efforts to approximate the system using simple models. Different community assembly pathways, for example, have been shown to lead to fundamentally different kinds of dynamic behaviour and long‐term steady states (Fukami, 2015), potentially requiring many different measurements and augmented models to characterise coexistence dynamics accurately (Letten & Stouffer, 2019). Moreover, many routine challenges that arise in empirical studies – e.g. transfer shock during invasion experiments, carry‐over effects from the environment in which young organisms are reared, or even germination failure or high mortality rates – are, in practice, exceedingly difficult to model without making resulting analyses intractably complicated.

An additional challenge is that real‐world systems are often highly diverse – including anywhere from dozens to thousands of species or taxonomic units even at the smallest possible observational scales (Jurburg *et al*., 2022). For diversity metrics that rely on measurements of species' monoculture performance, pairwise interactions, or invasion growth rates, this high diversity can necessitate impractically large numbers of experimental replicates [i.e. because the number of potential invasion pathways and interactions rise rapidly with community size; but see Song, Fortin & Gonzalez (2022), and Song (2025), for a discussion of efficient sampling strategies]. In systems that include many feasible steady states (e.g. Allee effects), this problem is magnified, as each of these states might need to be separately assessed as a potential local attractor (Song, Fukami & Saavedra, 2021). And, even when testing for global attractors, high diversity can present a challenge – e.g. even for relatively well‐understood classes of dynamic behaviour, building invasion graphs for communities that include more than a few dozen species is computationally challenging given current algorithms and computational performance (Hofbauer & Schreiber, 2022; Spaak & Schreiber, 2023).

### (2) Suggested workflow

Below, we describe a potential workflow for empirically assessing *if* and *when* species coexist based on the dynamic behaviour discussed in Section III.1, and the metrics of coexistence linking to these behaviours discussed in Section III.2. We note that determining *how* coexistence occurs often requires different metrics and experimental manipulations that are beyond the scope of this paper (see Section III.3).

#### (a) Select dynamic behaviours of interest and identify relevant metrics

The first step is to determine the specific ecological questions motivating your study and how they link to different classes of community dynamics behaviours, as discussed in Section III.1. For example, in more applied management settings, it might be most pertinent to ask, ‘for how long are species expected to persist?’ In this case, time‐to‐extinction analyses might be the optimal approach, even though they do not assess long‐term coexistence dynamics. Alternatively, in systems where conditions are relatively consistent over time, researchers might be interested in determining if coexistence is stable under small perturbations to the system. In these situations, analyses of feasible steady states or local attractors may offer a good balance between ease of application and breadth of insight. Finally, if interested in understanding coexistence under large perturbations, such as in systems with known periodic disturbance dynamics, invasion analysis may be able to reveal the existence of a global attractor, thereby providing especially robust and general insights about coexistence in the system.

**Fig. 4:**
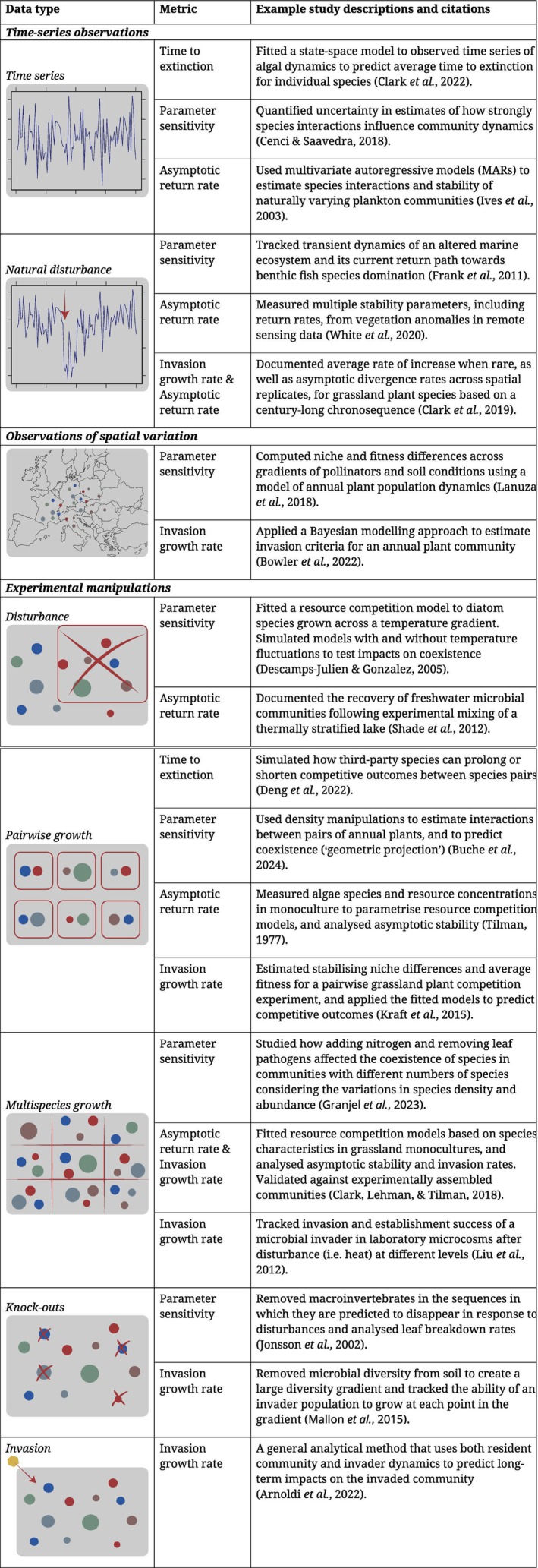
Linking data types and analytical methods for characterising coexistence in empirical contexts. Rows show different data types and the corresponding metrics used to assess coexistence in various published studies. Citations show example applications of each approach, with a short discussion of the work.

Once you successfully link from your ecological question to your mathematical class of coexistence behaviours, the next step is to determine your metric for characterising coexistence (Section III.2). There is a rough hierarchy to these metrics – e.g. time to extinction can be calculated for almost any kind of system but does not aid in understanding steady‐state dynamics or the community landscape of your study system (e.g. Figure 1). By contrast, invasion growth rates can, at least in theory, be used to characterise the overall global stability of coexistence in a system, but in exchange often require making very strong theoretical assumptions about the underlying dynamic model used to describe system dynamics. We emphasise that no single metric is ‘better’ or ‘more desirable’ than the rest – rather, each metric simply elucidates a different aspect of system behaviour, each of which is relevant for describing different facets of coexistence. Figure 4 provides empirical examples from the literature, where studies have identified and characterised coexistence, linking from a system‐specific question, to coexistence behaviour, to its associated metric.

#### (b) Link data and coexistence metrics

Coexistence metrics differ greatly in the kinds of data, theory, and model assumptions that are required to calculate them. Thus, it may be necessary to constrain the classes of dynamic behaviour that you choose to study based on the availability and types of data for your system (Fig. 4). For example, time series with smaller‐scale natural disturbances lend themselves most readily to asymptotic return rate and parameter‐sensitivity metrics. Most – but not all – metrics require researchers to build dynamic models, where empirical data are used to estimate parameters (e.g. density‐independent growth rates, species interaction coefficients). In doing so, it is critical to estimate uncertainty in parameter estimates properly and propagate this uncertainty into downstream calculations of coexistence (Bowler *et al*., 2022; Terry & Armitage, 2024). Further, it is important to acknowledge that parameter estimates – and especially estimates of species interaction coefficients – can be biased (Detto *et al*., 2019), including when sample sizes are small (Ives *et al*., 2003) and due to heterogeneity factors that often are excluded from the model – e.g. nutrients, microbial interactions, etc. (Rinella, Strong & Vermeire, 2020). Estimating species interaction strengths from observational data can be challenging due to a relatively small signal‐to‐noise ratio. Data with perturbations in species abundances, such as thinning experiments, planting species in pairs or multispecies communities across gradients of densities, or capitalising on natural disturbances or herbivory events, can therefore be helpful (Fig. 4).

We strongly advocate that multiple model structures should be compared to determine the best dynamic model, as coexistence metrics are often sensitive to model structure, even yielding different outcomes when applied to the same data set and when analysed using a single metric (Cervantes‐Loreto *et al*., 2023; Terry & Armitage, 2024). If a classical model structure, such as the general Lotka–Volterra equations, best fits the data, then applying many coexistence metrics becomes relatively simple. Parameter sensitivity and asymptotic return rates can be computed directly from species interaction coefficients (measured either from time‐series data or from pairwise interaction experiments), and even analysis of global attractors *via* permanence theory follows a relatively simple, established protocol. Alternatively, if theory suggests that interactions in the system are more complex – e.g. including Allee effects, non‐linear growth responses, or even higher order interactions – then analyses become more complicated, potentially limiting the kinds of behaviour that can be studied, and usually requiring larger quantities and different kinds of data (e.g. from multi‐species mixtures) (AlAdwani & Saavedra, 2022; Buche, Bartomeus & Godoy, 2024). At the far extreme, if you aim to avoid assuming an underlying dynamic model structure, then flexible data‐driven methods such as EDM or directly measuring invasion growth rates from experiments where species are systematically introduced into resident communities at low density may be especially useful (see Section III.2.*b*.*i*).

#### (c) Interpret your results

After using your data to calculate your metric of coexistence, you can determine if coexistence is expected in your system under the current set of environmental and biotic conditions. When interpreting results, keep in mind that theoretical models are, by definition, simplifications of complex ecological systems. As discussed in Section IV.1, many biologically important aspects of empirical systems tend not to be included in common classes of theoretical models (e.g. uncertainty in functional forms of biotic interactions, observation error, cross‐scale processes, etc.). Consequently, predictions about coexistence provided by your metric are inherently coupled with the assumptions used to produce them. As such, the assumptions and underlying models used to assess coexistence should always be clearly articulated in the results and interpretation.

#### (d) Validate coexistence predictions

We recommend researchers validate their predictions of coexistence (Tredennick *et al*., 2021; Yates *et al*., 2023). This is not a commonly considered part of current empirical workflows, but we strongly advocate that it should become the norm. Validation can be accomplished through multiple potential avenues. For example, out‐of‐sample validation can be readily used when data sets are sufficiently large; data are split into ‘training’ and ‘testing’ subsets, where model fitting occurs on the training data, and model predictions are assessed using the testing data. Applying this approach when examining coexistence using time‐series data presents a key challenge: the observation of species persistence over a given time period does not imply long‐term coexistence. Rather, in this case, observed changes in abundances can be compared to model predictions to assess if the dynamic model can correctly capture empirical time‐series patterns of abundance fluctuations. This can be challenging, as in many cases the dynamic models used to assess coexistence are fairly data hungry, and our data sets are relatively small. As such, an alternative approach is K‐fold cross‐validation, where the data is randomly split multiple times into training and testing subsets, and the model performance is assessed using the average of the out‐of‐sample predictions.

Studies of coexistence tend to focus on a small number of especially well‐studied systems (e.g. phytoplankton, annual grasslands). This provides exciting opportunities for further validating results by using independent data sets and experiments conducted in the same system. For example, drawing upon Fig. 4, a study that assesses coexistence using time‐series data could then compare predictions to outcomes from a disturbance, thinning manipulation, or species reintroduction if done in the same system. This would provide independent validation of model predictions and help address the concern that results are highly context dependent or only applicable under the current set of environmental and biotic conditions.

#### (e) Rinse and repeat

After interpreting results and comparing coexistence predictions to independent experiments for model validation, you may find that you need to collect more (or different) kinds of data, plan new experiments, develop alternate theoretical models, etc. Additionally, you may find that your results direct you to focus on different behaviours that require quantification of new metrics. We therefore strongly suggest an iterative approach to studying coexistence – i.e. returning to previous steps as needed to match your questions and underlying hypotheses, behaviours of interest, data availability, and practical needs.

### (3) Summary and outlook

Above all else, we remind readers that it is vital to define coexistence in terms of specific dynamic behaviours and metrics. We recommend that studies specifically state which behaviours and metrics they are applying (e.g. ‘we analysed asymptotic return rates to test for the existence of a feasible local attractor’), facilitating comparisons across studies of coexistence. Similarly, we urge researchers to remain humble and open‐minded when interpreting results from coexistence studies. In practice, there is no such thing as a single metric that is always right – both because metrics can only test for specific kinds of dynamic behaviour, and because these behaviours describe theoretical abstractions of complex empirical systems. To paraphrase the old adage about models: coexistence metrics will always be imperfect, but different metrics are useful under different circumstances.

We therefore suggest that future empirical studies of coexistence move away from single binary tests of whether a particular community can coexist or not, and instead towards applications of a diverse mixture of coexistence metrics that characterise more fully the system's dynamic behaviour. Similar shifts in scope away from individual metrics and towards holistic multidimensional tests have met with much success in general studies of ecological stability, yielding a complementary mix of different kinds of qualitative and quantitative understanding (Donohue *et al*., 2013; Domínguez‐García, Dakos & Kéfi, 2019; Radchuk *et al*., 2019; Medeiros *et al*., 2021; Allen‐Perkins *et al*., 2023). It is likely that the same insights apply to coexistence. For example, by quantifying both parameter sensitivity and asymptotic return rates, studies can account for the effect of perturbations on both parameters (e.g. growth rates, interaction coefficients) and state variables (e.g. species abundances; Medeiros *et al*., 2021). Similarly, jointly reporting asymptotic return rates and invasion growth rates within the same study can help show how coexistence is likely to respond to large *versus* small perturbations (Clark *et al*., 2019).

Additionally, we note that a little theory can go a long way – and making a few targeted theoretical assumptions can greatly reduce the scope of empirical data needed to test many coexistence hypotheses. For example, Hallett *et al*. (2019) used data from rainfall‐manipulation experiments to parameterise a demographic model – thereby enabling tests of coexistence along a global attractor with limited spatial and temporal replication. Nevertheless, it is also important to remember that if these *a priori* hypotheses, and corresponding assumptions, are poorly supported, then they can also lead to misleading conclusions. We therefore recommend explicit consideration and clear disclosure of these underlying assumptions, and assessment of the robustness of results when assumptions are not met. For example, impacts of demographic stochasticity are often assumed to be sufficiently small that they have minimal impacts on invasion success in tests of mutual invasibility. To test the impact of these small effects, Schreiber *et al*. (2023) reported the probability of invasion success averaged across a large number of replicates. Similarly, West & Shnerb (2022) show that even in highly complex systems, average predictions from a simple comparison of pairwise competition experiments correlated closely (but not perfectly) with those from more complex coexistence criteria. In general, it seems plausible that most classic coexistence metrics will, on average, produce qualitatively similar results when applied to empirical data even if all underlying assumptions are not met – so long as practitioners are careful to acknowledge that these results should be interpreted with care.

Lastly, we stress a balance between building on past hypotheses and models – which can significantly reduce the data requirements for analyses of coexistence – and testing alternative hypotheses and corresponding equations or even behaviours that might better fit the study system. For example, studies within the same location tend to use similar model structure, but across locations (e.g. Californian *versus* Western Australian annual plant systems), different dynamic models are usually assumed (e.g. Beverton–Holt *versus* Ricker model) (Levine & HilleRisLambers, 2009; Kraft *et al*., 2015; Wainwright *et al*., 2019; Bowler *et al*., 2022; Van Dyke *et al*., 2022). This aids in comparisons within the study site, but impedes inference about general, cross‐site patterns of coexistence, as different models – or even different methods for parameterising models – can yield opposing results (Cervantes‐Loreto *et al*., 2023). Simultaneously, however, Terry & Armitage (2024) noted that using the same model structure repeatedly impeded inferences of coexistence in some empirical settings, potentially leading to model‐specific dependencies of results. To help avoid this problem, combining insight from across multiple different theoretical frameworks (ideally representing multiple different dynamic behaviours and metrics) can help test the generality of results, and facilitate analysis both within and across ecosystems – e.g. by comparing predictions about coexistence across different sets of theoretical assumptions or environmental conditions (Ellner *et al*., 2019; Walker & Gilbert, 2023).

### (4) Future challenges

We believe that a pre‐eminent challenge for coexistence research in the coming years will be the development of new analytical methods that are built together with empirical applications. These new methods need not (and probably should not) seek to define new classes of dynamic behaviour (i.e. Section III.1) or even new coexistence metrics (Section III.2) – however, the current generation of methods for matching data to metrics and behaviours has been largely inherited from theoretical studies, and often leaves major gaps between theoretical assumptions and practical applications. In particular, these new methods should focus on making testable predictions that can be validated against available data (e.g. “will this species or community persist for the next X years?”), including long‐term observations, controlled experiments, and the growing body of proxy data coming from genetic, trait, and remote‐sensing studies (Borer *et al*., 2017; Gonzalez *et al*., 2023). Moreover, before new methods are introduced to a broader audience, they should be validated extensively against real‐world data to make their scope and limitations as clear as possible.

Another important next step in coexistence research will be to compare relationships between different coexistence metrics and dynamic behaviours in real‐world systems. While these relationships are well understood in theory, it is not yet clear how well these theoretical links apply in practice. For example, insights from studies of ecological stability suggest that multivariate relationships across different metrics are often much more constrained in empirical systems than is predicted by theory (Donohue *et al*., 2013; Radchuk *et al*., 2019). Interestingly, these constrained relationships could greatly simplify many analyses – e.g. if abundance dynamics for species with very long predicted times to extinction are, in practice, likely to be associated with feasible global attractors, then even relatively simple metrics could be used to test for complex dynamic behaviours.

Finally, an enduring challenge for coexistence research is to match both the spatial and temporal scales at which empirical data are available better to the scales that are most relevant for coexistence (Chesson, 2000b; Clark *et al*., 2019). Improving this link is critical under ongoing global change, which challenges many of the assumptions of classical coexistence theory. Strengthening this link will be important for validating new methods, assessing how well our mathematical theory translates to empirical predictions, and thus for using theory to make predictions that are directly useful for conservation, management, and restoration applications (HilleRisLambers *et al*., 2012; Hallett *et al*., 2023). Additionally, more general scaling approaches will be critical for identifying the spatial, temporal, and context‐based limits to our ability to understand and forecast coexistence (Maris *et al*., 2018; Tredennick *et al*., 2021).

## V. CONCLUSIONS

(1) Ecological coexistence has historically been described using a wide range of independently developed (and sometimes mutually exclusive) definitions and metrics.
(2) Most kinds of community dynamics fall into at least one of four classes, that themselves follow a nested hierarchy: feasible steady states (and invariant sets), feasible local attractors, feasible global attractors, or transient states.
(3) These four classes of behaviour can be used to define different aspects of ecological coexistence, and can be identified and tested using well‐established theoretical metrics: parameter sensitivity (for feasible steady states), asymptotic return rate (for feasible local attractors), invasion growth rates (for feasible global attractors), and time to extinction (in the case of transient states).
(4) Empirical systems present special challenges for studying ecological coexistence, e.g. due to incomplete biological understanding, impacts of observation error and stochasticity, the problem of scale, and other aspects of real‐world systems that violate common theoretical assumptions.
(5) Identifying and characterising ecological coexistence in empirical systems therefore requires careful consideration of the class of dynamic behaviour of interest, the kinds of metrics that can be applied to identify that behaviour, and the breadth of conclusions that can be drawn given available data and theoretical understanding.
(6) We stress that there is no single ‘correct’ class of behaviour or metric for defining and classifying coexistence – we therefore recommend that future studies take a more holistic approach, e.g. by assessing coexistence across a range of dynamic behaviours and metrics, and carefully articulating their scope and limitations.

## AUTHOR CONTRIBUTIONS

A. T. C. planned and wrote the first draft. S. J. S. proposed the structure for organising different classes of dynamic behaviours and metrics, with feedback from A. T. C. and L. G. S. All authors contributed significantly to planning, outlining, and writing the final text, and contributed significantly to revising the text and figs A. T. C. and L. G. S. then significantly revised the text to unify concepts, language, and style across sections. Lead authors for revising individual sections of the text include: G. B., O. G., and S. S. (parameter sensitivity); A. T. C. and C. K. (asymptotic stability and dynamic approaches); S. J. S. (invasion growth rates, permanence, and environmental and demographic stochasticity); L. G. S. (section on quantifying mechanisms); and A. T. C. (empirical challenges and workflow). C. K. designed the framework for Fig. 4 with feedback from A. T. C., R. G., O. G., and L. H.
